# The Management of Symptomatic Hydronephrosis in Pregnancy

**DOI:** 10.7759/cureus.52146

**Published:** 2024-01-11

**Authors:** Mohannad Hosny, Kimberley Chan, Mohamed Ibrahim, Vishali Sharma, Nikhil Vasdev

**Affiliations:** 1 Urology, Lister Hospital - East and North Hertfordshire NHS Trust, Stevenage, GBR

**Keywords:** ureteric stent, management, obstruction, pregnancy, hydronephrosis

## Abstract

The aim of this article is to provide a literature review on the management of symptomatic physiological hydronephrosis in pregnancy and compare different modalities of intervention when needed. In this review, we conducted an electronic literature search of peer-reviewed journal articles. The PubMed, Research Gate, and Google Scholar databases were queried with the following search terms: "pregnancy", "obstruction," and "hydronephrosis"; the terms "urolithiasis" and "kidney stone" were excluded. As a result, conservative treatment was successful and more favored for most of the patients and the clinicians in the different studies we found. Conservative management will usually include regular analgesia, positioning, and antibiotics. Close follow-up with ultrasound is always recommended. Intervention with ureteric stent insertion or nephrostomy tube insertion was less favored and only triggered by certain clinical criteria. In conclusion, symptomatic hydronephrosis in pregnancy can be safely treated conservatively. However, ureteral double-J stenting or percutaneous nephrostomy are effective and safe treatment methods in the minority of patients with persistent symptoms not responding to conservative management.

## Introduction and background

Pregnancy causes anatomical and physiological changes to the urinary system, in particular, the upper urinary tract. These changes can cause symptoms in some, and in some, it can also lead to pathological conditions. 

Physiologically, the increase in cardiac output during pregnancy is accompanied by a decrease in systemic vascular resistance. The renal blood flow increases by 30%, and the glomerular filtration rate is doubled [1]. This will cause a dilutional effect on the level of serum creatinine and urea. Clinically, a doubled level of creatinine can still be within the reference range for pregnant women.

Asymptomatic hydronephrosis during pregnancy can be found in more than 90% of pregnant women [2-4]. It becomes apparent in the second trimester, peaking between 24 and 28 weeks [5]. Usually, it is found in the right kidney and is mostly detected by ultrasound scan [6]. While the uterus is enlarging, during pregnancy it starts to cause extrinsic compression of the right ureter at the level crossing of the iliac vessels. The left ureter is relatively protected by the sigmoid colon and the uterine dextrorotation. Another cause for the liability of compression of the right ureter rather than the left is the crossing of the right ureter over the iliac vessels at the pelvic brim level, while the left crosses the vessels more proximally [7]. Constipation, which is common during pregnancy, is another cause for increased propensity to develop right-sided hydronephrosis. The increased volume of the sigmoid colon during constipation results in further dextrorotation of the gravid uterus with subsequent extrinsic compression of the right ureter. The dilated ureter and pelvicalyceal systems are more at risk of developing urinary infections with an increased risk of pyelonephritis up to 40% [8].

Postpartum hydronephrosis is markedly improved as compression from uterus is removed after birth [9]. In the literature, hydronephrosis during pregnancy is usually asymptomatic, with only an incidence of 0.2-3% where it becomes symptomatic [10-12]. Treatment of symptomatic hydronephrosis should not be delayed as it can lead to urosepsis, preterm labour or maternal/foetal death in the presence of infection [13]. Management of symptomatic hydronephrosis in pregnancy can be either conservative or surgical/radiological intervention. Conservative management consists of close observation and medical treatment, including intravenous fluids, good analgesia and oral/intravenous antibiotics if needed. In case of failure to control the symptoms with conservative management, surgical/radiological intervention is needed. Usually, the insertion of a ureteric stent or percutaneous nephrostomy will relieve the dilated and obstructed urinary tract [14]. Unfortunately, in the literature, it is not obvious and clear which is the best approach of treatment for pregnant women who are suffering from persistent symptomatic hydronephrosis with no causative disease, such as urinary stones or intrinsic obstruction.

The aim of this review was to try to find the optimum management method for these pregnant women complaining of pure gestational symptomatic hydronephrosis by running a wide search through different related studies. 

## Review

Materials and Methods

In this review, we made a comprehensive electronic literature search of peer-reviewed journal articles. The PubMed, Research Gate, and Google Scholar database was queried with the following search terms: "pregnancy", "obstruction", and "hydronephrosis"; the terms "urolithiasis" and "kidney stone" were excluded. We screened and assessed the eligibility of the identified articles. Then, we manually searched the selected articles to generate additional eligible citations. We consulted the international guidelines from the American College of Obstetricians and Gynaecologists [15], and the European Association of Urology [16]. Cardiovascular and Interventional Society of Europe Standards of Practice Committee [17] and Society of Interventional Radiology Safety and Health Committee [17]

Diagnostic evaluation

Ultrasound

Ultrasonography is considered a safe modality to evaluate the degree of hydronephrosis during pregnancy. It is also low-cost and gives immediate results with real-time capability [18]. Usually, ultrasound will show hydroureteronephrosis down to the pelvic brim. If the dilated ureter extends below that, distal ureteric stones can be considered [19].

Measurement of the ureteric jets on ultrasonography can enhance its characteristics in case of doubt. The jets are usually seen as effluxing urine from the ureteric orifice to the bladder. This is usually seen by a colour Doppler at the base of the bladder. However, ureteric jets cannot be seen in around 13% of patients even without obstruction, especially in the third trimester. These false-positive results can be decreased by doing the ultrasound to the pregnant patients in the contralateral decubitus position [20].

The renal colour Doppler ultrasonography can also measure the renal resistive index (RI). The RI indirectly measures the pressure inside the intrarenal vessels and the renal collecting system [21]. This pressure usually increases in acute ureteric obstruction. The RI is considered significant If the value is above 0.70 [22]. Delta RI (which is the difference between the RI in both kidneys) is better to be considered before planning intervention for symptomatic hydronephrosis in pregnancy [23]. Sometimes, patients' age and comorbidities, e.g., diabetes and hypertension, can cause an increase in the RI not related to urinary tract obstruction. For that reason, the clinical symptoms of the patients are extremely important in deciding the appropriate management.

Computed Tomography of the Kidneys, Ureters, and Bladder (CTKUB)

Non-contrast CTKUB is the standard investigation in non-pregnant patients for loin pain and renal colic. The sensitivity and specificity of this imaging modality is greater than 98% in detecting stones [24]. In our review, we have excluded stones from the criteria of search, but to rule out stones in pregnancy and confirm pregnancy-related hydronephrosis, this imaging modality is important. 

Radiation exposure with CT and nuclear medicine scans tends to be lower than the doses needed to harm the fetus. The American College of Obstetricians and Gynaecologists does not withhold doing CT scans on obstetric patients if required for a conclusive diagnosis [15].

The International Commission on Radiological Protection recommends that pregnant women should not be exposed to radiation more than 1 mGy [16], which is 2 mSv on the surface of pregnant women [25].

Teratogenic risks are mainly in the first trimester, which is why it is important to limit exposure to radiation at that time of conception. Compared to the third trimester, the radiation-causing teratogenic effects are much lower in the first trimester.

Non-contrast CT of the urinary tract usually uses a dose between 4.5 and 5 mSv. New techniques have been introduced and would utilise less radiation as low-dose (LD) and ultra-low doses, which would use lower than 3.5 and 1.9 mSv, respectively. Despite the reduction in the dose of radiation, these techniques would still have high accuracies as a diagnostic procedure with maintained high sensitivities and specificities [26].

Magnetic Resonance Imaging (MRI)

MRI does not use ionising radiation. This makes MRI scan safer to use during pregnancy as an alternative to ionising radiation methods. Intrauterine infants exposed multiple times to MRI scans after 20 weeks do not have abnormalities at the age of nine months [27]. Still, the safety of MRI in the first trimester has not yet been confirmed with enough evidence.

MRI can differentiate between physiological and pathological hydroureteronephrosis during pregnancy, with excellent results [28]. Usually, in physiological hydronephrosis of pregnancy, the level of obstruction is at the sacral promontory. The compression of the ureter happens between the psoas muscle and the gravid uterus. If the dilatation of the ureter extends beyond that point, a pathological obstruction should be suspected [29].

Management 

A literature review of different urology centres' experience in the management of symptomatic hydronephrosis (with the exclusion of urolithiasis) was performed (Table 1). 

**Table 1:** Comparing different studies on the management of symptomatic hydronephrosis in pregnancy WBC - white blood cells; CRP - C-reactive protein

Study and sample size	Date	Study design	Summary of study	Summary of findings
Puskar et al. [30] N=103	2001	Retrospective study	Study evaluating the conservative management of symptomatic hydronephrosis and the need of intervention in case or progression to urosepsis	Conservative management was successful in 94% of patient and only 6% who progressed to urosepsis needed ureteric stenting, which improved their condition.
Fainaru et al. [12] N=56	2002	Retrospective study	A study evaluating the success rate of conservative management in symptomatic hydronephrosis. Study was done between January 1998 and June 2001.	Conservative management was successful in 92% of patients of the study. Only four patients needed ureteric stent insertion for the progression of their symptoms clinically or radiologically on an ultrasound scan.
Tsai et al. [14] N=93	2007	Retrospective study	Comparative study between the management of symptomatic hydronephrosis with conservative versus ureteric stenting. Study was done between January 2000 and December 2004.	Conservative management was successful in 21/25 patients. 4/25 managed conservatively needed stent insertion. The 25 patients who had stent inserted were all symptoms free except four only who complained of loin discomfort.
Chitale et al. [35] N=1750	2010	Prospective study	Pregnant women with symptomatic physiological hydronephrosis whose pain was refractory to routine enteral or parenteral analgesia over 72 hours were offered postural drainage to evaluate if this helps with analgesic control.	7.4% had refractory pain to routine analgesia. 93.1% of such patients had symptomatic improvement with postural drainage in a semi-prone position in bed, with the affected side facing upward and non-dependent.
Cecen et al. [31] N=53	2014	Retrospective study	All pregnant women with symptomatic physiological hydronephrosis were offered double-J stent insertion. Patients could decline the offer and have a trial of conservative management. The study evaluated the effectiveness of the two modalities of treatment. Study was done between 2004 and 2013.	45% accepted intervention with double-J stent insertion, while 55% insisted on conservative treatment. There were no complications recorded for patients who had double-J stent insertion. No patients who had conservative management required further surgical intervention.
Ercil et al. [32] N=211	2017	Retrospective study	Symptomatic pregnant women were investigated for the parameters that may decide the best suitable method of treatment. Study was done between 2011 and 2016.	Around 62% of pregnant women in the second and third trimesters were managed conservatively. Intervention with ureteric stent was done for the rest guided by persistent WBC and CRP mainly.
Simsir et al. [36] N=84	2018	Retrospective study	Retrospective evaluation of the success rate of percutaneous nephrostomy and double J stenting in the treatment of symptomatic, physiological maternal hydronephrosis. Study was done between January 2000 and December 2016	Percutaneous nephrostomy was favoured over double J stenting. Patients who had double J stenting had a higher rate of second intervention (p=0.0018) and the time to secondary intervention was also significantly earlier (p=0.0025).
Saylam et al. [33] N=102	2021	Retrospective study	Retrospective evaluation of the clinical course and management of pregnant women with maternal physiological hydronephrosis, followed up between July 2017 - February 2020.	Conservative management was successful in (96.1%) of patients, (3.9%) ended up with acute pyelonephritis and needed intervention with ureteric stent insertion under local anaesthesia. All had a normal vaginal delivery.
Demir M et al., [34] N=227	2021	Retrospective study	Comparing different parameters in two groups of patients with symptomatic hydronephrosis managed conservatively and with ureteric stents. Study was done between December 2010 and December 2020.	Surgical insertion of stent was needed in patients with more symptoms of infection, renal dilatation on ultrasound scan. The recommended a cut off for the dilatation of the renal pelvis for the intervention during the first two trimester and the last trimester

Conservative Management Versus Intervention

Many studies have shown that the majority of symptomatic hydronephrosis can be treated conservatively and that surgical intervention should only be reserved for patients who do not improve, present with signs of infection or with additional obstructive disease. 

In a study of 3400 pregnant women, 103 women were found to have severe symptoms related to their upper urinary tract. Patients with renal or ureteral stones were excluded from the study. Starting from the first visit, all patients were assessed by doing serial blood tests, including full blood count and renal functions, urine analysis and ultrasonography on a monthly basis till one month after delivery. For patients diagnosed with acute pyelonephritis, the tests were done more frequently till the resolution of symptoms. Initially, all patients were offered conservative management (analgesia, hydration and positioning). Antibiotics were offered only to patients diagnosed with acute pyelonephritis. Ninety-seven (94%) of the patients responded well to the conservative management. Only 6% had a progression of symptoms to urosepsis and needed ureteric stenting. Stents were inserted under local anaesthesia and ultrasound guidance. This resulted in rapid improvement of their symptoms and normalisation of their blood and urine parameters within a few days. The stents were removed after one month from delivery with no complications. The study concluded that ureteric stenting might be necessary in a minimal number of patients whose symptoms progress despite conservative management of their symptomatic hydronephrosis in pregnancy [30].

In a three-and-half-year retrospective study, data was collected from 56 pregnant women admitted with symptomatic hydronephrosis to a prenatal care unit. That study showed conservative management was successful in 92.2% of the patients. They were managed with analgesia, intravenous fluids and antibiotics. The pregnant women were assessed with renal ultrasound scans, urine and blood tests, including blood pictures, and renal function tests. Only four patients did not respond to the conservative management and showed clinical progression of their symptoms as progression to sepsis, renal function test deterioration or evidence of ureteric flow obstruction on ultrasonography. These patients were managed by cystoscopic insertion of stent under ultrasound guidance and intravenous sedation. They all delivered at term, except one of the four patients who had a stent was induced at 34 weeks due to psychiatric indication. The stents were removed within four to six weeks from delivery without complications. The study concluded that most of the symptomatic hydronephrosis in pregnancy respond well to conservative management, and if ureteric stent insertion is needed, it should be a safe and efficient option of management [12]. 

A comparative study conducted in a four-year interval was done on 93 patients with symptomatic hydronephrosis admitted to a department of obstetrics and gynaecology. Fifty patients had moderate to severe hydronephrosis on ultrasound scans; the rest with mild hydronephrosis were excluded from the study. They were managed randomly with ureteric stent versus conservative management. Half of the patients had a stent inserted, and the other half were managed conservatively. Only five out of the conservatively managed did not respond well to analgesia, hydration and antibiotics. They ended up having ureteric stent insertion. They defined failure of conservative management in patients with deteriorating renal functions or non-resolving infection after 48 hours of intravenous antibiotics. Stent insertion was done under local anaesthesia. The treated patients with the stent had instant relief of their symptoms, and only four patients experienced stent symptoms in the form of loin pain and suprapubic discomfort. The stents were removed one month after delivery. The study concluded that ureteric stenting for symptomatic hydronephrosis is an effective modality of treatment but always needs to be the second choice after conservative management because of its complications and postoperative-associated discomfort [14].

In another nine-year retrospective study, 53 pregnant women diagnosed with hydronephrosis were all offered the insertion of double-J stents. Twenty-four (45%) women ended up having a ureteric stent inserted, and 29 (55%) were adamant on having only conservative management. No serious complications were seen during or after stent insertion. The conservatively managed group did not need any intervention. Antibiotics and analgesics were used to overcome signs of infection in the conservatively managed group and were successful. There was no significant difference in the pain score for the conservative and the stented groups at one week after first admission and six weeks post-delivery (p>0.05). All the patients had their delivery after the 37th week. This study concluded that ureteric stent insertion for symptomatic hydronephrosis in pregnancy does not add a benefit over conservative management. Intervention should be only used for complicated cases or pathological obstruction [31].

In a five-year study on 211 pregnant women with symptomatic hydronephrosis where stone disease was excluded. They investigated the parameters that may decide the most suitable method of treatment for these women. They divided their results according to the pregnancy trimester. One hundred four patients in the second trimester and 107 in the third trimester. They managed to treat conservatively 65 (62%) patients out of the 104 from the second-trimester group and 66 (61%) out of 107 patients from the third-trimester group. The conservative management was intravenous fluid and analgesia. Antibiotics were used only used if patients developed fever and showed high white blood cells (WBC) and C-reactive protein (CRP). Patients who failed the conservative management by showing persistence of their fever and/or WBC and CRP despite medical treatment ended up having ureteric stent insertion. Stents were cystoscopically inserted under ultrasound guidance. The parameters they compared were patient age, hospital stay, urine culture positivity, renal functions, pre and post-treatment pain score, degree of hydronephrosis, WCB and CRP level. They found that the significant parameters for the surgically treated group during their second trimester were CRP and WBC levels, preterm labour rate and pre-treatment pain, but the rate of fever was higher in the conservatively managed group. The same results were in the third-trimester group, but the pyuria was higher in the conservatively managed third-trimester group. They concluded that clinical signs such as degree of hydronephrosis and extent of pain might guide the clinician towards the appropriate management for symptomatic hydronephrosis. The need for intervention is mainly related to the persistent rise of WBC and CRP [32].

A retrospective study was done on 102 patients presenting with acute symptomatic hydronephrosis during their pregnancy, where urolithiasis was excluded. In all patients, basic investigations, including blood tests (serum creatinine levels, white blood cell count), urinalysis and culture together with renal US, were performed in their first visit. These were repeated at least once a month until one month postpartum. Conservative management included positioning and analgesics. Intravenous antibiotics were only used if a patient showed signs of infection. It was successful in 98 (96.1%) out of 102 patients. Only four patients developed acute pyelonephritis and had an increased degree of hydronephrosis on ultrasound scans. They ended up having ureteric stent insertion under local anaesthesia. The signs of infection subsided within four days. Post stent insertion, they complained of frequency, haematuria, and loin pain. The four of them had normal vaginal delivery at an average of 39 weeks. Their stents were removed one month after delivery with no complications [33].

In a ten-year retrospective analysis of 227 pregnant women visiting the clinic due to symptomatic hydronephrosis. They divided their patients into two groups. One hundred thirty-three patients were managed conservatively, and 94 patients needed treatment with ureteric stent insertion. They did a comparison between different parameters for the two groups, including age, gestational week, renal functions, blood count and ultrasonographic findings. They found that renal function impairment, severity of infection, degree of hydronephrosis and renal pelvis anterior-posterior (AP) diameter were significantly higher in the group who needed the intervention. The rest of the parameters, including age, gestational week, and blood test, did not differ significantly. They concluded that early surgical intervention is better in patients who do not respond well to conservative management. Intervention is needed if the renal pelvis AP diameter is greater than 16.5 mm in the first and second trimesters but can be up to 27.5 mm till intervention is needed in the third trimester [34]. 

Apart from conventional conservative methods mentioned before, a study was found to use mainly postural drainage of the kidney as an effective way of managing symptomatic hydronephrosis during pregnancy. In that study, they placed their patients in a semi-prone position in bed while the painful side was facing upward in a non-dependant position. They also raised the head end of the bed 10 degrees during the management period. One hundred twenty-one (93%) out of 130 patients clinically improved, and they did not even need regular analgesia, and none of them needed any intervention as well. They followed them up with an ultrasound scan three months after delivery, and this showed a resolution of the hydronephrosis. Therefore, this manoeuvre can be particularly useful for patients who remain symptomatic despite regular analgesia and can help prevent the need for invasive uro-radiological intervention [35]. 

Percutaneous Nephrostomy (PCN) Versus Double-J Stent Insertion

We found one retrospective study of 84 patients comparing the rate of success of PCN and double-J insertion as management of symptomatic hydronephrosis during pregnancy. The study showed that PCN was preferred over double-J insertion because of the lower re-intervention rate. In that study, they used ultrasound scans for the diagnosis and the grading of hydronephrosis. The grading was done according to the Society of Foetal Ultrasound. They divided the patients into two groups according to the way the hydronephrosis was managed. Group A had PCN (38 patients), and Group B had ureteric stent insertion (46 patients). Any patients diagnosed with urinary stones or pelvi-uretic junction obstruction were excluded from the study. The option of having a PCN or a stent was according to the patients' preference. If the patient had no specific preference, then the choice was made according to the surgeon and some predictive factors. Factors in favour of PCN included high-grade hydronephrosis, ultrasound features of infection, patients at high risk for anaesthesia and early gestational weeks. In case these findings were not found, a stent insertion was preferred, mainly if the patient was in the third trimester and the risk of anaesthesia was low. The study also compared and analysed the time of re-interventions between PCN versus double-J insertion. It concluded that PCN was more effective and feasible than Double-J insertion as a treatment for symptomatic hydronephrosis in pregnancy, especially since PCN needs less reintervention with a longer time between reintervention, compared to double-J stent insertion [36].

## Conclusions

Hydronephrosis in pregnancy is a common anatomical and physiological change. Although most pregnant women are asymptomatic with it, some can have persistent severe pain, fulminant pyelonephritis or urosepsis. It often poses both diagnostic and treatment dilemmas for the urologists diagnostically in trying to limit radiation exposure in this cohort and from a treatment perspective on when to intervene and intervention types.

On review of the existing literature, it is widely accepted that conservative management should be the first line where appropriate. Some studies recommended early intervention for those in whom there were serious risks to the mother or foetus due to an obstructed kidney. In terms of choice of surgical intervention, our review shows that ureteric stenting using ultrasound guidance under local anaesthesia or sedation is a safe option with rapid relief of renal obstruction and patient’s symptoms. A single study favoured nephrostomy insertion due to the requirement for less reintervention; however, consideration should be given to the challenges of managing a long-term nephrostomy during pregnancy. 

In conclusion, conservative management should be considered the first choice whenever possible for managing symptomatic hydronephrosis in pregnancy. However, if clinical parameters require surgical intervention, ureteric stenting or nephrostomy insertion are considered safe management options.
